# GDM as a unique pathophysiological entity or a transitional pregnancy-induced glucose metabolism abnormality identifying primary diabetes types?

**DOI:** 10.3389/fphys.2026.1778006

**Published:** 2026-02-27

**Authors:** Vendula Bartáková, Eliška Ambrožová, Petr Žák, Kateřina Kaňková

**Affiliations:** 1 Department of Pathophysiology, Faculty of Medicine, Masaryk University, Brno, Czechia; 2 Second Department of Internal Medicine, Faculty of Medicine, Masaryk University, Brno, Czechia

**Keywords:** diabetes in pregnancy, gestational diabetes mellitus, glucose metabolism abnormality, metabolic programming, postpartum diabetes risk

## Abstract

Gestational diabetes mellitus is defined as glucose intolerance first diagnosed during pregnancy; however, this diagnosis may actually represent a heterogeneous spectrum of primary diabetes phenotypes unmasked by the metabolic stress of pregnancy rather than a single pathophysiological entity. The current review aims to summarise available knowledge about the diabetes spectrum in pregnancy with particular focus on its pathophysiology, risk factors and postpartum destiny. Another aim was to discuss possibilities for stratification of the women according to their immediate and future risks of postpartum persistence of glucose intolerance and its complications in later life. Specific objectives of the paper are: (1) to summarise data on physiological metabolic changes in pregnancy, (2) characterise the diabetes spectrum in pregnancy, (3) address the current state of the art in GDM diagnosis and management, (4) to summarize data on postnatal development and maturation of the infants who experienced GDM *in utero* and, finally (5) discuss possibilities to stratify GDM women according to the later risk for persistence of glucose intolerance after delivery.

## Introduction

Diabetes is the most common complication of pregnancy nowadays and is routinely considered–unless it pre-exists pregnancy (typically as type 1 diabetes - T1DM) – as gestational diabetes mellitus (GDM) although pregnancy can unmask a latent predisposition to type 2 diabetes mellitus (T2DM) or maturity-onset diabetes of the young (MODY) or accelerate the development of T1DM or latent autoimmune diabetes of adults (LADA). Pregnancy is, therefore, a metabolic test of a kind, and the presence of any type of diabetes has a significant impact on the future metabolic health of the affected woman.

GDM has been defined as hyperglycaemia with an onset or its first recognition during pregnancy since the end of 1970s (Classification and diagnosis of diabetes mellitus and other cate gories of glucose intolerance, 1979). Later definitions added another time-specific piece of information: that GDM usually resolves after delivery (Association AD, 2019). The majority of recent definitions thus strictly distinguish between GDM as a glucose metabolism abnormality that resolves after pregnancy and different forms of diabetes that first manifest in pregnancy and have not been diagnosed before, i.e., T1DM, LADA, T2DM, or MODY, in which glucose metabolism abnormality typically persists after pregnancy. Eventual postpartum persistence of glucose abnormality in women with a GDM history should always be re-evaluated. Ideally, right after puerperium, by oral glucose tolerance test (oGTT); however, patients’ compliance with this recommendation is notoriously rather low, due to many factors interfering with the timing and logistics in this extremely challenging period of life. Published worldwide data indicate participation in postpartum testing between 20% and 50% (Bellamy et al., 2009; Ferrara et al., 2009; Keely, 2012), suggesting that we have rather incomplete information on the postpartum metabolic situation, indeed. At the same time, several studies, including an earlier one from our group, documented non-negligible persistence of postpartum glucose abnormality in women diagnosed with GDM. It is, therefore, plausible that the diagnosis of GDM is overrepresented in real settings and in a certain proportion of subjects, we are in fact dealing with the primary forms of diabetes. Both GDM onset and manifestation of primary diabetes are triggered by the same factors specific to pregnancy (for details, see further). Given the low postpartum oGTT participation, metabolic abnormalities might persist unmanaged for a long time after delivery and exert significant glucotoxic damage to tissues.

The current review aims to summarise published data supporting our hypothesis that GDM could be a much rarer condition than perceived and, at least in some cases, represents a transitional pathophysiological state identifying primary diabetes types. Specific objectives of the paper are: (Classification and diagnosis of diabetes mellitus and other cate gories of glucose intolerance, 1979): to summarise data on physiological metabolic changes in pregnancy, (Association AD, 2019), characterise the diabetes spectrum in pregnancy, (Bellamy et al., 2009), review the current state of the art in GDM diagnosis and management and, finally, (Ferrara et al., 2009), discuss approaches to stratify women according to the later risk of persistence of glucose intolerance after delivery.

### Physiology and pathophysiology of metabolic changes in pregnancy

Insulin, an anabolic hormone released by the β-cells of the Langerhans’ islets in the pancreas, modulates glucose homeostasis by stimulating glucose uptake into peripheral tissues, inhibiting hepatic glucose production, and suppressing the release of stored lipids from adipose tissue. Insulin resistance is a state in which normal concentrations of insulin fail to achieve an appropriate biological response downstream of the insulin receptor. As a result, the β-cells must release more insulin than usual to regulate blood glucose levels.

Pregnancy (in its later phase) is associated with insulin resistance and hyperinsulinemia that may predispose some women to develop/exacerbate diabetes. The etiopathogenesis of GDM is still incompletely understood. While increased insulin sensitivity is typical for the first phase of physiological gestation, insulin resistance normally develops in the second and third trimester, as a consequence of rising concentrations of maternal hormones (such as oestrogen, progesterone and leptin) and placental hormones (cortisol, prolactin, human placental lactogen and growth hormone) that counteract insulin action (Newbern and Freemark, 2011). The decrease in insulin sensitivity by approximately 50% is comparable to that observed in T2DM, and subsequent hyperinsulinemia is believed to be beneficial for foetal growth through the facilitation of nutrient transport to the foetus (Catalano, 2014).

Insulin resistance may develop earlier in women with pre-existing impairment of glucose metabolism (Nadal et al., 2009). Additionally, insulin signalling is affected by impaired phosphorylation of the insulin receptor substrate 1 (IRS-1) or the insulin receptor, although the number of receptors on the cell surface remains the same (Barbour et al., 2007). Such development of insulin resistance is promoted by pro-inflammatory cytokines such as tumour necrosis factor α (TNF-α), interleukin 1 (IL-1), and interleukin 6 (IL-6), which affect insulin signalling by inhibiting IRS-1 through serine phosphorylation (Santangelo et al., 2019). This is more likely to occur in individuals with inflammatory comorbidities and obesity. Mitochondrial dysfunction, oxidative stress, and endoplasmic reticulum stress are well-known consequences of gluco- and lipotoxicity, affecting insulin synthesis, secretion, and β-cell survival (Moyce and Dolinsky, 2018). Furthermore, pro-inflammatory cytokines can induce β-cell de-differentiation and increase endoplasmic reticulum stress (Nordmann et al., 2017).

The ability of β-cells to compensate for the physiological increase in insulin resistance by a matched increase in insulin secretion determines glucose tolerance. Healthy women can increase insulin secretion and compensate for this, whereas women with a latent defect in insulin secretion or reduced β-cell reserve cannot. This inability later manifests as GDM. The resulting abnormal metabolic situation during GDM pregnancy adversely influences the metabolic status of the child, both antenatally and perinatally, as well as after birth. Apart from established metabolic abnormalities assessed by routine laboratory assays, there are a host of recently identified disturbances (such as an altered spectrum of adipokines, cytokines, etc.) potentially contributing to the adverse consequences for both mother and baby (Lacroix et al., 2013).

Further understanding of the pathophysiology of GDM and the mechanisms of foetal programming induced by GDM and diabetes in general is definitely warranted to allow for risk stratification of the population of pregnant women. However, establishing causality is challenging in humans and epidemiological studies, which are often complicated by multiple confounding factors and are very demanding. Several experimental animal models have been developed, but they have not yet been able to capture the full complexity of GDM (Alejandro et al., 2020).

### Spectrum of glucose homeostasis disorders in pregnancy

According to World Health Organisation (WHO) and the International Federation of Gynaecology and Obstetrics (FIGO), hyperglycaemia in pregnancy (HIP) can be classified as either pre-gestational diabetes, GDM or diabetes in pregnancy (DIP) (Organization WH. World Health Organization, 2013; Hod et al., 2015).

Pre-gestational diabetes includes women with known T1DM, T2DM or rarer forms of diabetes (for example, MODY). GDM may occur anytime during the antenatal period and is not expected to persist postpartum (Immanuel and Simmons, 2017). DIP applies to pregnant women with hyperglycaemia who were first diagnosed during pregnancy and meet the WHO criteria of diabetes in the non-pregnant state (Guariguata et al., 2014). For a clearer summary, see Table 1.

**TABLE 1:** Terminology of hyperglycaemic statuses in pregnancy.

Terminology	Meaning
HIP	Any form of hyperglycaemia, first diagnosed in pregnancy, as T2DM, T1DM, MODY, LADA, GDM, DIP or any other form of pre-diabetes, including persistent hyperglycaemia after delivery
DIP	Obvious diabetes in pregnancy according to WHO classification, first diagnosed in pregnancy
GDM	A form of hyperglycaemia, first diagnosed in pregnancy, normalised after delivery
early GDM	Hyperglycaemia first detected during pregnancy before the 24th week of gestation
late GDM	Hyperglycaemia first detected during pregnancy at 24th week of pregnancy or later

The International Diabetes Federation (IDF) estimated that 21.1 million (16.7%) of live births to women in 2021 had some form of HIP. Of these, 80.3% were due to GDM, while 10.6% were the result of diabetes detected prior to pregnancy, and 9.1% due to DIP (IDF Diabetes Atlas Diabetes Atlas, 2017). The American Diabetes Association (ADA) estimates that most (75%–90%) cases of HIP are GDM (Association, 2014). Some studies suggest that the proportion of GDM may actually be lower and of other forms higher.

Based on current data, the focus should definitely be given to MODY manifestation during pregnancy, since it is probably not such an exceptional diagnosis and up to 5% of patients diagnosed as GDM may actually be MODY cases, specifically the glucokinase subtype of MODY (GCK-MODY, also MODY2), because there is a lack of evidence of other, much rarer subtypes of MODY in pregnancy. Accurate diagnosis of MODY is crucial for appropriate management, treatment decisions and fetal monitoring. Women suspected of having GCK-MODY had statistically higher odds of delivering neonates below the 25th percentile for weight if the child inherited the mutation and the mother is insulin-treated during pregnancy, but, opposite, there is a risk of macrosomia and severe postpartum hypoglycaemia in a foetus that did not inherit the GCK mutation and whose mother is not properly treated (Lima Ferreira et al., 2021; Hattersley et al., 1998; Majewska et al., 2023). Unfortunately, MODY is often initially misdiagnosed as a different type of diabetes, even in non-pregnant patients (up to 90% of cases), and the journey to a correct diagnosis often takes several months or even years. Thus, its recognition during a relatively short period of pregnancy is a diagnostic challenge (Ługowski et al., 2025). Typical signs for diagnosis of GCK-MODY are body mass index (BMI) < 25 kg/m2 and fasting glucose ≥5.5 mmol/L, in addition to positive family medical history of diabetes and small glucose increase during oGTT, however, the definitive diagnosis requires genetic testing (Chakera et al., 2014; Ellard et al., 2000; Murphy et al., 2008). In Table 2, you can find a summary of key indicators for the diagnosis of GCK-MODY (Mohamed et al., 2014).

**TABLE 2:** Key indicators for GCK-MODY diagnosis.

Positive family history	Diabetes present across multiple generations
Early onset	Typically before age 25 (but can be later)
Mild/Asymptomatic Presentation	Blood sugar levels might be only mildly elevated
Lack of obesity and insulin resistance	Both are typical for T2DM
Lack of specific autoantibodies	The presence of specific autoantibodies is typical for T1DM

Regarding other specific types of diabetes, there are only a few references in the literature to pancreatogenic diabetes during pregnancy, for example, in women with pancreatic adenocarcinoma or acute pancreatitis, since those are extremely rare cases (Quaresima et al., 2021).

There are also very few studies evaluating the impact of antenatal corticosteroid therapy on maternal glycaemia. Those show that corticosteroids worsen hyperglycaemia in most pregnant women, regardless of pre-existing disturbances of glucose metabolism, and may therefore induce transient steroid-related hyperglycaemia (Satyaraddi et al., 2024; Gopal et al., 2023).

As concerning GDM, it is important that two subtypes are sometimes described, which may differ in terms of both causes and consequences - the early subtype and the late subtype. Early GDM refers to hyperglycaemia first detected anytime before the 24th week of gestation, and in many women, this reflects pre-existing disturbances of glucose metabolism. In contrast, late GDM is diagnosed at the 24th week and later and predominantly results from the progressive physiological insulin resistance during the second and third trimester. Early GDM is associated with a higher likelihood of persistent dysglycaemia or overt diabetes postpartum, whereas late GDM more often resolves after delivery, although both confer a long-term risk of type 2 diabetes (Zaccara et al., 2022).

### GDM - from epidemiology to treatment

#### Epidemiology and diagnostic criteria

The incidence of GDM is rising worldwide; it is now considered to be the most common medical complication during pregnancy. For example, in the United States, the GDM prevalence range has increased from 6.9% in 2019 to 8.0% in 2023 and in Europe from 5.4% in 2016 to 7.8% in 2021 (Anastasiou et al., 2020; Wang et al., 2022). This is happening for multiple reasons (described below), and screening programs contribute to increasing cumulative prevalence, no matter what diagnostic criteria are used (Reece et al., 2009).

The diagnostic criteria for GDM vary and remain controversial, complicating the comparison of research data. In 2017 in most countries, there has been a move towards the more strict diagnostic criteria advocated by the International Association of the Diabetes and Pregnancy Study Groups (IADPSG)/WHO (Metzger et al., 2010; Saeedi et al., 2021) and this has resulted in a general increase in the overall prevalence of GDM (Song et al., 2018). According to these recommendations, an oGTT is performed by measuring the plasma glucose concentration while fasting and then one and 2 hours after ingesting 75-g of glucose. But, for example, in the United States, GDM is most commonly diagnosed using a two-step approach, with a non-fasting 50-g glucose challenge test followed, only if abnormal, by a 100 g 3-h oGTT, where at least two abnormal values are required; the one-step 75-g oGTT using IADPSG criteria (≥1 abnormal value) is used less often (Committee ADAPP, 2024). In Australia and New Zealand, new, higher cut-offs for positive oGTTs are recommended from 2025 onwards, with the aim of reducing overdiagnosis in women at lower risk and focusing specifically on women with a higher likelihood of complications (Sweeting et al., 2025). Table 3 shows recommendations and GDM criteria used in different parts of the world.

**TABLE 3:** Guidelines for the diagnosis of GDM and other recommended monitoring during and after pregnancy in different countries.

	Glycaemia in the first trimester of pregnancy or first prenatal visit	oGTT between 24–28 weeks of pregnancy					oGTT after pregnancy with WHO criteria
Organisation/Country		FPG	Glucose challenge	1-h	2-h	3-h	
WHO 1999* (Organization, 1999)		≥7.0	75 g oGTT	—	≥7.8	—	4–12 weeks postpartum
IADPSG/WHO 2013 most used worldwide * (Metzger et al., 2010)	FPG in first prenatal visit (≥5.1 mmol/L abnormal)	≥5.1	75 g oGTT	≥10.0	≥8.5	—	4–12 weeks postpartum
American Congress of Obstetricians and Gynecologists** (AuthorAnonymous, 2011)	FPG in high-risk women (≥5.3 mmol/L abnormal)	≥5.3	100 g oGTT	≥10.0	≥8.6	≥7.8	4–12 weeks postpartum
Canadian Diabetes Association** (CDA, 2008)	FPG in first trimester (≥7.0 mmol/L abnormal)	≥5.3	75 g oGTT	≥10.6	≥8.9	—	6–24 weeks postpartum, before planning another pregnancy and every 3 years
United Kingdom*** (Landi et al., 2019)	-	≥5.6	75 g oGTT	-	≥7.8	—	only optional FPG 6–13 weeks postpartum
Australia, New Zealand (based on ADIPS) (Sweeting et al., 2025)	oGTT if HbA1c is 6.0–6.4% + anamnesis of previous GDM	5.3–6.9	75 g oGTT	≥10.6	9,0–11,0	—	6–12 weeks postpartum
China* (based on IADPSG) (Chen et al., 2022)	FPG in first prenatal visit (≥5.6 mmol/L abnormal)	≥5.1	75 g oGTT	≥10.0	≥8.5	—	4–12 weeks postpartum
India**** (Welfare MoHaF, 2018)	75 g oGTT in first prenatal visit, only 2-h plasma glucose (≥7.8 mmol/L abnormal)	—	75 g oGTT	—	≥7.8	—	6 weeks postpartum

*One value in 2^nd^ trimester oGTT, is sufficient for GDM, diagnosis.

**Two or more values in 2nd trimester oGTT, are required for GDM, diagnosis.

***only selective testing based on risk factors (previous GDM, BMI, above 30 kg/m^2^.

previous macrosomic baby, family history of diabetes (in first-degree relative) and ethnicity with a high prevalence of diabetes).

****Regardless of whether the women are fasting or not at the beginning of the oGTT.

#### Risk factors for developing GDM

Higher BMI before pregnancy, older maternal age, positive family history, previous occurrence of GDM and history of large for gestational age babies are all considered as crucial risk factors for developing GDM. Studies further suggest the contribution of additional factors, including polycystic ovary syndrome, multigravidity, increased weight gain during pregnancy, higher glycaemic variability, a sedentary lifestyle, smoking and pre-existing hypertension (Plows et al., 2018; Ustianowski et al., 2023). Multiple studies have focused on non-modifiable factors including genetics. Several associations were ascertained between GDM and variants in genes for insulin receptor, glucokinase, melatonin receptor, potassium channel, hepatocyte nuclear factor, peroxisome proliferator receptor, etc. Also, fat mass and obesity associated genes were studied in this context. Most of those genes were associated with T2DM too, which underlies the hypothesis of a shared genetic basis for GDM and T2DM (Kwak et al., 2012).

Metabolomics has been used in pregnancy to assess metabolic profiles by measuring numerous low-molecular-weight metabolites. However, studies on amino acids, free fatty acids and conventional metabolites show inconsistent results in GDM, although women with higher insulin resistance and hyperglycaemia exhibit metabolic changes, similar to non-pregnant insulin-resistant individuals (Alesi et al., 2021).

Maternal diet is an important non-genetic risk factor for GDM(Tobias et al., 2012) not only in quantitative terms (influencing body weight and composition), but also qualitative differences in food intake may contribute to the development of GDM and thus constitute a modifiable environmental risk factor (Bartáková et al., 2018). Diet is also a powerful modulator of the gut microbiota, whose impact on insulin resistance and the inflammatory response in the host is well known. Changes in the gut microbiota composition in the sense of reduced diversity have been described in pregnancies either before the onset of GDM or after its diagnosis (Koren et al., 2012). It remains unclear if the changes in the gut microbiota contribute to or are a consequence of the development of GDM. Finally, the microbiota of GDM patients can be transmitted to the offspring and colonisation before birth by specific taxa associated with GDM occurs (Wang et al., 2018).

Whether there are other sensitive markers that could be identified ans exploited for diagnostic purposes using more complex individual-level data such as omics, and if these can feasibly be implemented in clinical practice remains unknown and will be important to consider in future studies (Benham et al., 2023).

#### Treatment

Lifestyle modification recommendations and diabetic diet are the first-line strategies in the management of GDM(49). These are very similar to those recommended for patients with T2DM, but regarding the incentive of pregnancy and foetal nutrition requirements, dietary arrangements are not so strict. The emphasis is on optimising diet composition and limiting foods with a high glycaemic index, rather than on strict carbohydrate or caloric restriction. Similarly, physical activity must be of a reasonable intensity and character.

Regular self-monitoring of glucose levels is a very important integral part of the treatment strategy for all patients with diabetes, including women with GDM. Typically, glucose levels are measured in capillary blood using a blood glucose meter. Nowadays, continuous glucose monitoring (CGM), which provides patients with real-time feedback on glucose levels from interstitial fluid, has become a standard in developed countries. It significantly improves adherence among patients with diabetes, which correlates with better health outcomes (Battarbee et al., 2024). A randomised controlled trial found that the use of CGM reduced the incidence of baby macrosomia (Yu et al., 2014), but another one found no significant difference in the large infant risk between CGM and self-monitored glucose in women with gestational and pre-pregnancy diabetes (Voormolen et al., 2018). So, for now, there is limited data on the effectiveness of CGM for improving pregnancy outcomes in women with GDM, but experience in T1DM patients suggests that CGM could also be beneficial for other types of diabetes, including GDM. The limitation of CGM’s wide application is its higher cost, as well as the potential for users to encounter difficulties interpreting CGM data, underscoring the need for individualised care approaches.

Pharmacotherapy of diabetes in pregnancy is, in general, limited by the lack of safety data for the majority of oral antidiabetic drugs. There is also variability in clinical practice regarding the choice of first-line drug and the initiation of GDM treatment when diet and lifestyle changes are insufficient. Studies show that maternal characteristics, including body mass index (BMI)≥30 kg/m2, family history of T2DM, prior history of GDM and higher glycated haemoglobin (HbA1c), increased the likelihood of the need for insulin treatment (Alvarez-Silvares et al., 2022). The selected insulin or insulin analogue, along with its dosing frequency and dosage, is tailored to the measured glucose profile. Over the last years, metformin has been increasingly used for GDM treatment (as a substitute for insulin) and has shown comparable or even better results in maternal or perinatal outcomes (Picón-César et al., 2021; Spaulonci et al., 2013). Since metformin crosses the placenta, its effects on fetal and childhood growth are uncertain. Some studies suggest no difference in growth and developmental outcomes between children of mothers treated with metformin versus insulin; on the other hand, a systematic review found that metformin-exposed foetuses showed accelerated postnatal growth, leading to higher childhood BMI, which is linked to adverse cardiometabolic outcomes (Picón-César et al., 2021). There is, therefore, a strong need for further research on the long-term effects of intrauterine exposure to metformin.

Additional drugs with a potential for use in pregnancy are glyburide/glibenclamide, which belongs to the group of sulfonylurea-based antidiabetic drugs and has a low rate of crossing the placenta. The few studies conducted in GDM do not yet fully agree on its non-inferiority to insulin and metformin (Hebert et al., 2009; Balsells et al., 2015; Sénat et al., 2018). Recently, inositol, which transfers glucose into cells for conversion into fatty acids, has been investigated. If used early in pregnancy, it can reduce the likelihood of gestational diabetes in at-risk pregnant women by improving insulin sensitivity and glycaemic control (Fabio et al., 2023). However, there is no evidence to date that it can be used directly to treat GDM. Vitamin D deficiency in pregnant women is common throughout the world and the detection rate of GDM is positively correlated with the degree of vitamin D deficiency (Holick, 2007). It has been confirmed that additional vitamin D supplementation can improve hyperglycaemia and delay, or even prevent, the progression of diabetes. Also, a clinical trial conducted in women with GDM who received vitamin D supplementation showed a tendency toward improved glycemic control, but results were not statistically significant. While the preliminary findings are promising, further research is needed to establish definitive conclusions. Also, the modulation of the gut microbiota by dietary interventions during pregnancy is an emerging area of interest, given the potential effects on maternal and, consequently, neonatal health (Ponzo et al., 2019; Gohir et al., 2015). Probiotics are a relatively new intervention; they target mothers’ metabolism, and can reduce blood sugar levels, enhance insulin sensitivity, prevent gestational diabetes and reduce the maternal and foetal complications resulting from it (Benhalima et al., 2019). Using probiotics to modulate the gut microbiota may become part of a comprehensive treatment (de Albuquerque et al., 2024).

#### Postnatal development and maturation of the infants who experienced GDM *in utero*

GDM is associated not only with maternal but also with neonatal adverse outcomes and represents a significant risk for the offspring. During the perinatal period, GDM increased the incidence of macrosomia, congenital malformations, premature delivery, respiratory distress syndrome during delivery, neonatal hyperbilirubinemia or hypoglycaemia and low Apgar score, both in the first and fifth minutes. Moreover, in childhood, slow psychomotor development was observed, with delays affecting speech, social reactions and motor skills (Kowalczyk et al., 2002; Rizzo et al., 1995). GDM is suspected to impacts offspring neurodevelopmental and cognitive outcomes. An increased risk of deficit/hyperactivity disorder (ADHD) was detected in a meta-analysis (Zhao et al., 2019).

There is increasing epidemiological evidence linking the early-life environmental exposures (i.e., maternal malnutrition/overnutrition, environmental chemicals, stress) and corresponding pathophysiological changes (circulating mediators, neuro-hormonal changes, low-grade inflammation, etc.) with later-life health outcomes–conceptualised as the ‘developmental origins of health and disease’ (DOHaD) paradigm. Compelling studies from animal models have provided strong evidence in support of the DOHaD concept. These have, for example, shown that *in utero* exposure to maternal diabetes and/or obesity disrupts the development and function of the hypothalamus, predisposing offspring to obesity (Chu and Godfrey, 2020). The actual mediators of DOHaD are numerous and are being explored. For instance, long-term effects on offspring may be mediated by epigenetic changes, i.e., through the regulation of gene activity without changes in the DNA sequence. Epigenetic changes occur, for example, in the form of histone modifications, DNA methylation, or the disruption of the function of non-coding RNAs, including microRNAs, with DNA methylation being the most studied mechanism to date (Dłuski et al., 2021). In pregnant women with GDM, epigenetic studies have shown altered gene methylation and chromatin modifications in the baby’s DNA, providing a potential substrate for disrupted gene regulation (Salbaum and Kappen, 2012). However, further follow-up will be necessary to determine the long-term effects of GDM on offspring of women diagnosed with GDM during pregnancy.

Offspring of GDM mothers are, in any case, more susceptible to suffering from cardiovascular diseases (CVD) and childhood obesity or to developing T2DM more frequently in subsequent life (Metzger et al., 2010; Metzger et al., 2008). Some studies also show a higher risk of allergic diseases or other immunodeficient diseases (Chu and Godfrey, 2020). For an overview, see Table 4.

**TABLE 4:** Development and maturation of the infants who experienced GDM *in utero*.

Period of offspring´s life	Adverse outcomes
*Prenatal*	macrosomia, congenital malformations
*Perinatal*	premature delivery, respiratory distress syndrome during delivery, neonatal hyperbilirubinemia, hypoglycaemia, low Apgar score, shoulder dystokia
*Childhood*	slow psychomotor development (motor skills, speech, social reactions), ADHD
*Long-term*	obesity, type 2 diabetes, cardiovascular diseases, allergy, autoimmune diseases

#### Postpartum outcome of GDM women and possibilities to stratify GDM according to the later cardio-metabolic risk

Women with GDM have an increased risk of not only adverse perinatal outcomes (i.e., during pregnancy up to 1 year postpartum) such as hypertension or preeclampsia, complications during delivery, difficult breastfeeding, etc., but also of persistence of abnormalities in glucose metabolism postpartum. They confer a 7× higher likelihood of developing any glucose metabolism abnormality (especially prediabetes or T2DM in the future; T2DM is expected to develop in 20%–50% of these women within 10–20 years (Bellamy et al., 2009; Bartáková et al., 2015). Several studies aimed at the persistence of abnormalities in glucose metabolism postpartum, respectively early after GDM pregnancy, briefly, 15%–20% of women with GDM complicated pregnancy had T2DM or impaired glucose tolerance (IGT) (Benhalima et al., 2019). Studies focusing on other forms of diabetes developing after a GDM pregnancy are rare. For example, our study with 305 GDM women showed 16.7% prevalence of any form of glucose metabolism abnormality; of those, 62.7% had prediabetes, 37.3% had manifest diabetes (31.4% were diagnosed as T2DM, 5.9% as T1DM) (Bartáková et al., 2015); another study has shown the prevalence of T1DM after a GDM pregnancy 7% (Füchtenbusch et al., 1997).

The risk of a persisting abnormality of glucose metabolism after delivery is not the same for every woman with GDM (White et al., 2020). Factors such as fasting glucose levels, BMI, HbA1c, the total number of abnormal glucose values during antenatal oGTT, and the timing of diagnosis (early or late GDM) are important in predicting postnatal glucose intolerance (Gupta et al., 2024). Some studies indicate a higher fasting plasma glucose during pregnancy oGTT test and lower BMI in those GDM women, who later developed T1DM (Unnikrishnan et al., 2016) and, opposite, higher BMI and higher levels of glucose in 120th min of oGTT in pregnancy in those with T2DM development in their later life (Cypryk et al., 2005). The risk could also reflect likely etiopathogenetic heterogeneity of the disease (e.g., various contributions of genetic, environmental and other factors). On the contrary, breastfeeding has been shown to significantly lower the risk of developing postnatal diabetes both in children and women with a history of GDM (Taylor et al., 2005). So far, it seems that the longer and more intensive exclusive breastfeeding, the greater the reduction of the risk of diabetes outbreaks (Eades et al., 2024).

Predicting postnatal diabetes after GDM is now feasible through various risk prediction models, which utilise a combination of clinical data, glucose test results and medical history. Many of which, according to their authors, show relatively good discriminatory power (AUC 0.725–0.940). The variables that appear most frequently in the models are age, BMI, family history, physical activity levels, ethnicity, insulin requirement in the therapy, etc., whereas the most significant appear to be blood sugar values in oGTT and HbA1c (Köhler et al., 2016; Parkhi et al., 2023; Lee et al., 2007). The addition of lipid markers could significantly improve the reclassification of patients as well as early postpartum data (e.g., glycemia, weight) (Lappas et al., 2015; Belsti et al., 2024). Interestingly, polygenic risk scores for T2DM are associated with an incidence of T2DM after GDM, but they provide only a modest improvement in discrimination and risk reclassification, suggesting limited incremental value for routine clinical risk stratification at present (Choi et al., 2024). However, most models suffer from significant methodological limitations, namely, insufficient description of predictor selection, limited internal as well as external validation, leading to a generally higher risk of bias, etc. So, challenges remain in standardising these tools, and there is a need for further research and external validation to make them applicable for widespread clinical use (Belsti et al., 2024). Further examination of the metabolome and its incorporation into predictive models, together with the routine evaluation of additional biomarkers in the first trimester of pregnancy - such as antibodies used for T1DM diagnosis or the measurement of leptin, adiponectin, and resistin (Kapustin et al., 2020) - might improve the prediction of future disorders of glucose metabolism; however, their clinical significance is yet to be clearly established (Atarod et al., 2020; Sun et al., 2024; Huhtala et al., 2023; Liu et al., 2024; Semnani-Azad et al., 2024).

By identifying women with GDM who are at higher risk of persisting some form of abnormality of glucose metabolism early postpartum, we can implement earlier intervention and ensure more intense postpartum follow-up. Effective lifestyle modifications, especially dietary changes, physical activity, maintaining a normal BMI, and breastfeeding, can significantly lower the chances of developing DM (Ikoh Rph and Tang Tinong, 2023). So, there is a strong need for repeated check-ups and personalised education, including targeted interventions (Elomrani et al., 2025). Unfortunately, the participation of GDM women in postpartum follow-up programs is still unsatisfactory–more than half of patients did not pass a recommended OGTT test after delivery (Ferrara et al., 2009; Keely, 2012), and moreover, longer follow-up studies for GDM women and their children are rare.

## Conclusion

Pregnancy is often described as a “window” to future health (Catov and Margerison-Zilko, 2016) since the physiologic hormonal, metabolic, cardiovascular and other changes that occur during this time act as a natural “stress test” for the body’s adaptation capacity. The majority of women in the developed countries receive rather intense medical attention and even specialised care during their pregnancy, which makes this an ideal time for risk assessment of diabetes persistence risk and for eventual subsequent management and personalised follow-up. Should we apply the definition of GDM rigorously as a glucose metabolism disorder occurring exclusively during pregnancy and disappearing after delivery, the prevalence would likely be lower and primary types of diabetes would be considered more often. Unfortunately, we can reassess the classification only ex post (after delivery) and a significant proportion of data is missing at this moment. Yet, the stratification of pregnant women at the time of routine GDM testing is appealing since personalised treatment and follow-up could yield considerable benefits in terms of morbidity, quality of life, nd healthcare spending in the future.
